# Essential Role of Complement in Pregnancy: From Implantation to Parturition and Beyond

**DOI:** 10.3389/fimmu.2020.01681

**Published:** 2020-07-31

**Authors:** Guillermina Girardi, Joshua J. Lingo, Sherry D. Fleming, Jean F. Regal

**Affiliations:** ^1^Department of Basic Medical Sciences, College of Medicine, Member of QU Health, Qatar University, Doha, Qatar; ^2^Division of Biology, Kansas State University, Manhattan, KS, United States; ^3^Department of Biomedical Sciences, University of Minnesota Medical School, Duluth, MN, United States

**Keywords:** innate immunity, complement, pregnancy, preeclampsia, preterm birth, pregnancy loss, fetal development

## Abstract

The complement cascade was identified over 100 years ago, yet investigation of its role in pregnancy remains an area of intense research. Complement inhibitors at the maternal-fetal interface prevent inappropriate complement activation to protect the fetus. However, this versatile proteolytic cascade also favorably influences numerous stages of pregnancy, including implantation, fetal development, and labor. Inappropriate complement activation in pregnancy can have adverse lifelong sequelae for both mother and child. This review summarizes the current understanding of complement activation during all stages of pregnancy. In addition, consequences of complement dysregulation during adverse pregnancy outcomes from miscarriage, preeclampsia, and pre-term birth are examined. Finally, future research directions into complement activation during pregnancy are considered.

## Introduction

The complement system or alexin was identified more than 100 years ago by Jules Bordet for its ability to “complement” the role of heat stable antibody in protecting the host and lysing bacteria (1). Since then numerous activities of this powerful enzymatic amplification cascade have been extended beyond host defense and immunopathology to defining a role for the complement system in homeostasis and normal development. Once viewed as simply a humoral component of the immune system, the functions of complement have been recognized far beyond an extracellular system that lyses bacteria. This review will focus on the role of complement in helping to orchestrate a normal pregnancy, and the evidence that control of the system is important to prevent pathology in the mother and rejection of the semi-allogeneic fetus. In addition, control of the complement system is essential for normal placental and fetal development to avoid lifelong adverse consequences for offspring of those pregnancies. We will provide background on the complement system, as well as an evaluation of the literature to date that shows a role for complement in the following events: pre-implantation, implantation, and placental development, as well as normal development of the fetus to parturition and labor. Studies demonstrating dysregulation of the complement system in recurrent pregnancy loss, preterm birth, preeclampsia, hypertensive disorders of pregnancy, and intrauterine growth restriction will be reviewed. Clearly, problems in pregnancy can lead to adverse effects in offspring of that pregnancy. Thus, we will extend our discussion to the role of the complement system in neurodevelopmental and behavioral disorders as well as the risk of cardiovascular and metabolic disease in the offspring following adverse pregnancy outcomes.

## Immunity vs. Reproductive Success

To guarantee survival and maximize reproductive success, resources are subject to trade-offs within the organism (2, 3). A successful pregnancy requires lengthy periods of time and considerable amounts of energy. In addition, the fetus demands additional energy from the mother; therefore, the utilization of resources need to be adapted during gestation. The assignment of available resources to reproduction occurs at the expense of other systems like immune function (4). Reproduction results in reduced immunity, and conversely, infection, and immune responses reduce reproductive success. A successful pregnancy requires the development of a maternal inflammatory reaction that is thought to control exaggerated fetal demands. However, an excessive inflammatory reaction has been associated with adverse reproductive outcomes. The complement system, part of the innate immune response, plays a crucial role in normal pregnancy from conception to delivery (5). However, uncontrolled complement activation results in several pregnancy complications such as miscarriage, preeclampsia and preterm birth (6, 7). This is in agreement with the trade-off paradigm; increased immune responsiveness restricts reproduction.

## The Complement System: Extracellular and Intracellular

### Extracellular

**Figure 1:**
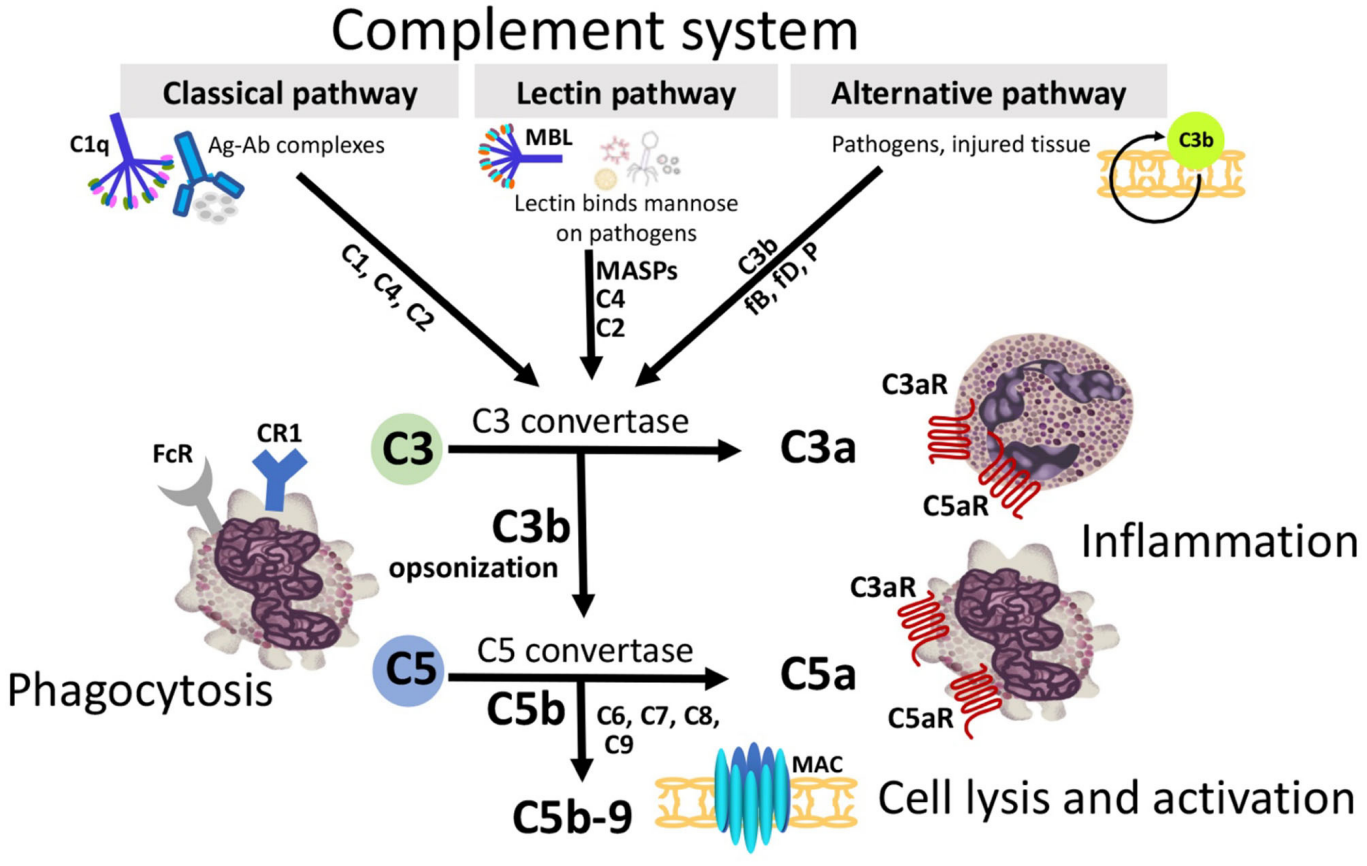
Three extracellular complement initiation pathways culminate in a common terminal pathway. Grey boxes identify initiation and terminal pathways with complement components identified along the arrows. The Classical pathway is activated by antigen/antibody complexes, recognized by C1q in complex with C1r and C1s. Proteases C1r and C1s cleave C4 and C2 to generate the Classical pathway C3 convertase C4b2a. The Lectin pathway is triggered by binding of mannose-binding lectin (MBL) or ficolins to carbohydrates on the target membrane. The MBL-associated serine proteases (MASPs) then cleave C4 and C2 generating the C3-convertase C4b2a. The Alternative pathway, an amplification loop, is triggered when the C3b protein directly binds a microbe, foreign material, or damaged tissue. C3b also binds factor B (fB) to form C3bB. FB is cleaved by factor D (fD) to form Alternative pathway C3-convertase, C3bBb. This convertase is stabilized by properdin (P). C3b opsonizes targets for phagocytosis and B-cell activation. All 3 initiation pathways converge on C3 with distinct C3 convertases which cleave C3 to generate the anaphylatoxin C3a, and more C3b to form the C5-convertases (C4b2a3b and C3bBb3b). C5-convertase then cleaves C5 into C5a and C5b. C3a and C5a can attract and activate inflammatory cells and contract smooth muscle through receptors (C3aR, C5aR1, and C5aR2). C5b binds C6, C7, C8, and multiple copies of C9 forming the membrane attack complex (MAC) complex. MAC pores can cause cell death by osmotic flux.

**Figure 2:**
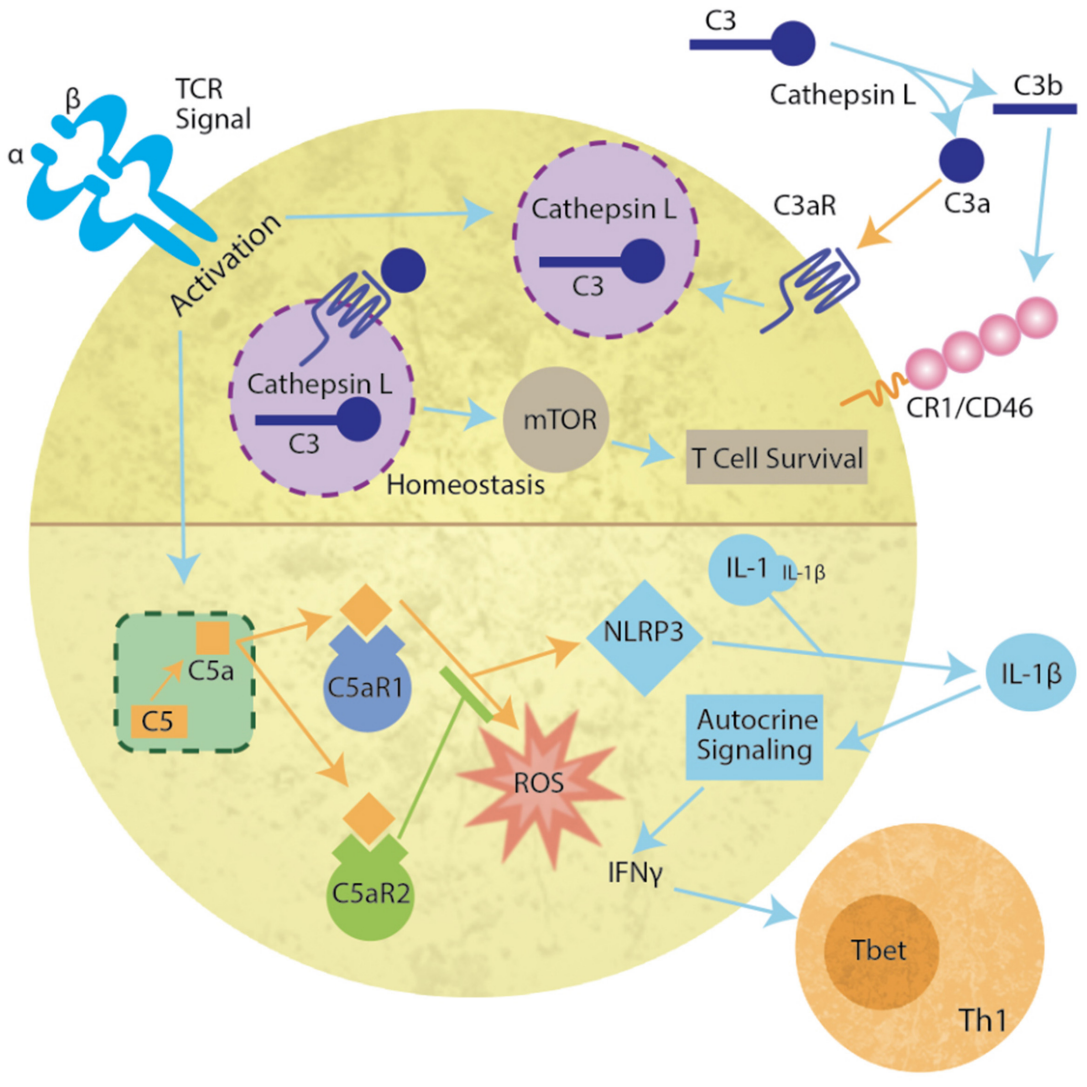
Intracellular complosome activation in the T cell. During homeostasis, lysosomal Cathepsin L cleaves C3 to C3b and C3a. C3a binds to C3aR on the vesicular membrane to activate mTOR resulting in T cell survival (upper panel). Cognate antigen engagement by T cells results in lysosome fusion with the membrane, release of a Cathepsin L and C3 containing vesicle resulting in extracellular Cathepsin L-mediated cleavage of C3. C3b binds complement receptor 1 or CD46 (MCP) and C3a binds C3a receptor (C3aR) (Upper right) inducing T cell proliferation and differentiation. In addition, upon TCR activation, intracellular C5 is cleaved by an unknown protease allowing C5a to bind either intracellular C5aR1 or C5aR2 (lower half of diagram). C5aR1 induces reactive oxygen production (ROS) and NLRP3. NLRP3 cleaves pro-interleukin 1 (IL-1) producing IL-1β. The autocrine signaling induces IFNγ release and Th1 T cell differentiation.

Jules Bordet's concept of complement has been elucidated over the years to reveal the intricacies of complement activation, regulation, and clinical significance. A brief overview of the cascade and its inhibitors are discussed below. Complement can be divided into three extracellular pathways (Figure 1) and an intracellular pathway (Figure 2) that differ in activation and inhibition.

The first pathway to be fully characterized was rightfully coined the Classical complement pathway. The Classical pathway is initiated by a single IgM or multiple IgGs complexed to antigen or a pathogen-associated molecular pattern (PAMP). The antibodies then change conformation to expose a binding site for the first protein in the classical cascade, C1 (8). C1 is composed of C1q, the antibody binding molecule, and C1r_2_s_2_, initially inactive serine proteases. C1q recognition of the antibody/antigen complex induces a conformational change in C1r, transforming it to an active state which activates C1s (9, 10). Once both serine proteases are activated, the zymogenic cascade works quickly, cleaving C4 and C2. The cleavage products form the complex C4b2a, the C3 convertase. As the name suggests, C4b2a then cleaves C3 into C3a, an anaphylatoxin, and C3b which joins C4b2a forming the C5 convertase, C4b2a3b. As the previous pattern suggests, the newly formed C5 convertase cleaves C5 into C5a, another anaphylatoxin, and C5b, the initiator of the Membrane Attack Complex (MAC). Occurring even more quickly than the aforementioned processes, C6 joins the deposited C5b, which is quickly followed by C7, C8, and an amplified quantity of C9 to form a well-designed, yet asymmetrical pore that leads to targeted lysis of the pathogen (8, 11).

Outside of the Classical pathway, C1q also further modulates the immune system and plays a role in development (12). Upon binding the cC1qR on an immature dendritic cell, C1q induces NF-κB translocation to the nucleus and the successive production of IL-10, IL-12, and TNFα followed by dendritic cell maturation (13). Additionally, C1q has an antiproliferative effect on T cells and other peripheral blood cells, with the exception of erythrocytes (14). These data suggest that C1q may be critical to tolerance of peripheral antigens. C1q also functions in angiogenesis and the clearance of apoptotic cells (12).

Fifty years later, another complement cascade was discovered by Pillemer et al. (15). Pillemer proposed an Alternative pathway which was not formally accepted until almost a decade later (8). A unique feature of the Alternative pathway is autoactivation through the hydrolysis of a disulfide bond on a complete C3 protein to form C3(H_2_O) (16). C3(H_2_O) then binds and changes the conformation of Factor B in a Mg^2+^-dependent manner (17). The serum protein, Factor D cleaves the altered Factor B, and the Bb fragment remains associated with C3(H_2_O) to form the Alternative pathway C3 convertase, C3bBb. This complex is stabilized by another serum protein, Properdin (18), and proceeds to cleave another molecule of C3 forming the Alternative pathway C5 convertase, C3bBbC3b (19). The Alternative pathway C5 convertase also cleaves C5 to C5a and C5b. Despite the striking differences in molecular composition of the convertases, both pathways proceed down the same terminal pathway after cleavage of C5 to form the MAC. Due to the antigen-independence of the Alternative pathway, it is often considered to function as an amplification loop for the Classical pathway or the next extracellular pathway we will discuss, the Lectin pathway.

The third extracellular complement activation pathway was discovered nearly 40 years later (20). While remarkably similar to the Classical pathway, the Lectin pathway instead activates the complement cascade through pattern recognition molecules [either Mannose-Binding Lectin (MBL) or a ficolin] that recognize monosaccharides exposing 3′ and 4′ hydroxyl groups, such as glucose, mannose, and *N*-acetyl-glucosamine (10, 21). Either MBL or a ficolin will engage with an array of monosaccharides in a similar manner to C1q-IgM recognition. The Lectin pathway proteases, MASP-1 and MASP-2, are activated sequentially with MASP-1 autoactivation when MBL binds the target carbohydrate, and subsequent activation of MASP-2. While there are differences between studies, under physiological conditions, the cascade continues by either MASP-1 or 2 cleaving C2 and MASP-2 cleaving C4 prior to the rest of the zymogenic cascade following along the same path as the Classical pathway (10, 22).

### Intracellular

More recently, an intracellular system was identified and primarily characterized in human CD4+ T cells (23, 24). Since this initial discovery of the “complosome,” many non-immune cells have been identified as containing functional intracellular complement components, including mesenchymal stem cells (25), intestinal epithelial cells (26), and pancreatic β cells (27). The complosome (Figure 2) not only includes intracellular C3, but CD4+ T cells also contain a C3a receptor (C3aR) on the lysosome and Cathepsin L in the lysosome. Cathepsin L constitutively cleaves C3 into C3a and C3b. It is proposed that the intracellular membrane C3aR and C5aR signal similar to vesicular signaling by other G protein coupled receptors (GPCR) (28, 29). Importantly, the signals produced by vesicular receptors may differ from those on the plasma membrane (30). During homeostasis, the C3a-C3aR system sustains low-level mTOR activity, thereby promoting T cell survival *in vivo* (24, 29). However, upon TCR activation, the intracellular C3 system that is normally confined to the lysosome, translocates to the plasma membrane, allowing extracellular release of both C3a and C3b and signaling through their membrane-bound receptors, C3aR and CD46 (MCP) or CR1. This induces IFNγ production, and the development of T_H_1 cells (31). In addition, upon TCR engagement, intracellular C5 is cleaved by an unknown protease (23). Activation of the intracellular C5a receptor (C5aR1) increases production of reactive oxygen species (ROS) and induces the NLRP3 inflammasome. The formation of the inflammasome induces cleavage of IL-1, producing IL-1β that signals in an autocrine fashion to increase IFNγ. However, C5aR2 can also bind C5a to negatively regulate NLRP3 inflammasome activity and reduce the Type 1 response produced by intracellular C5a (32). Intracellular complement systems have also been implicated in intestinal damage during ischemia/reperfusion events (26). These data are particularly critical at the highly vascular interface between the mother and the fetus as prenatal hypoxia has severe implications for neonate cognition and development (33).

### Regulation

**Figure 3:**
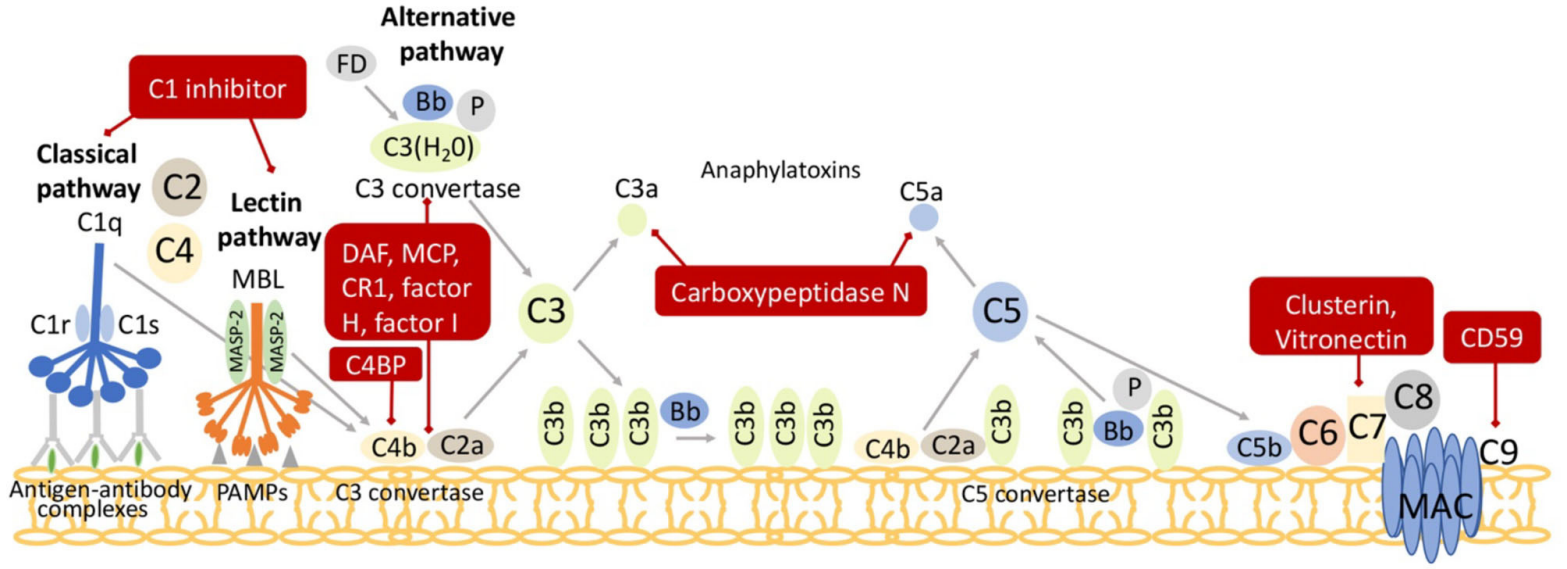
Regulators of the complement pathway. Both surface and soluble complement regulatory molecules identified in the red boxes inhibit critical junctures of the cascade and degrade C3 convertases (C4b2a or C3bBb) or C5 convertases (C4b2a3b or C3bBbC3b) and the anaphylatoxins C3a and C5a. C1 inhibitor prevents excessive activation of both Classical and Lectin pathways. The Classical and Lectin pathway C3 convertase, C4b2a, is regulated by complement receptor 1 (CR1), C4 binding protein (C4BP), decay accelerating factor (DAF), membrane cofactor protein (MCP), and factor I. The Alternative pathway C3-convertase, C3bBb, is regulated by CR1, factor I, factor H, DAF, and MCP. The anaphylatoxins, C3a and C5a, are degraded by carboxypeptidase N. Finally, Vitronectin and Clusterin inhibit the C5b-8 complex while CD59 (protectin) inhibits C9 insertion into membrane attack complex (MAC).

Considering the zymogenic nature of the complement cascade and the risk for self-activation, it is easy to understand why multiple complement regulatory molecules evolved. Two main regulatory systems to control complement activation have been identified: membrane bound regulators and soluble regulators (Figure 3). A subset of membrane bound regulators are quite effective in accelerating decay of the C3 convertase. Decay Accelerating Factor (DAF; CD55) affects convertases of both the Classical and Alternative pathway. DAF is globally expressed on many somatic cells and functions to protect them from complement activation (8, 34). Similarly, complement receptor 1 (CR1) also inhibits the Classical and Alternative Pathway. Unlike DAF, CR1 is expressed mostly on antigen presenting cells, erythrocytes, and phagocytes. CR1 functions as a cofactor for Factor I, discussed below. With limited expression in mice, membrane cofactor protein (MCP), otherwise known as CD46, functions similarly to CR1 as a Factor I cofactor. However, MCP specifically aids the degradation of C3b bound by protein rather than those bound by other acceptor molecules (35). Additional membrane bound regulators prevent the formation of the MAC and attenuate target cell damage. Vitronectin, clusterin, and CD59 (protectin) prevent the culminating step of all three pathways. Vitronectin and clusterin inhibit insertion of the C5b-7 complex or C7 and C8, respectively into the MAC (36, 37), while CD59 prevents the insertion of C9 into the membrane. CD59 is expressed on nearly every cell in the human body (38) and stops the complement cascade at C5b-8, saving somatic cells from inattentive MAC formation. C4-binding protein (C4BP) inhibits the enzymatic activity of the Classical C3 convertase, C4b2a (39), while Factor H is a soluble cofactor that competes with Factor B for binding to C3b in the Alternative pathway. In conjunction with co-factors [C4 binding protein (C4BP), Factor H, MCP, and CR1], Factor I regulates all three extracellular pathways by cleaving C3b and C4b and preventing the formation of active C3 and C5 convertases. Another soluble regulator is the anaphylatoxin inactivator, plasma Carboxypeptidase N. Carboxypeptidase N cleaves a terminal arginine from C3a and C5a to generate their des Arg derivatives, C3a des Arg and C5a des Arg, altering their biological activities and potency at the C3aR and C5aR (8, 40).

### Complement Regulation at the Maternal-Fetal Interface Is Essential for a Favorable Pregnancy Outcome

The maternal-fetal interface is rich in complement inhibitors, suggesting they have evolved to protect the placenta and control complement activation (41), hence preventing adverse pregnancy outcomes. In humans, DAF (CD55) and MCP (CD46) control C3 activation early on in the complement cascade, whereas CD59 acts in the terminal pathway to prevent formation of MAC (42). In mice and rats, an additional widely distributed complement regulator not found in other species is produced. It is known as Complement receptor 1 related protein y (Crry) (43). Crry is structurally similar to MCP and DAF, with complement inhibitory activities similar to CR1 (44). Molina and colleagues deleted the gene encoding Crry in mice and discovered that homozygous Crry^−/−^ mice died *in utero* (43). In Crry^−/−^ mice, C3 deposited on the embryo and in the ectoplacental cone, suggesting that the absence of Crry resulted in complement-mediated embryonic death. Breeding Crry heterozygotes (Crry^+/−^) with mice deficient in C3 to generate Crry^−/−^ on a C3 deficient background rescued the pregnancies confirming that uncontrolled complement activation was responsible for loss of Crry^−/−^ embryos. Importantly Crry^−/−^ mice on the C3 deficient background survived gestation and were born healthy (43). In addition, treatment of the BPH mouse strain that exhibits high blood pressure and frequent fetal loss with Crry targeted to placental C3b deposition resulted in a decrease in placental inflammation and increased favorable pregnancy outcomes (45). Together these studies demonstrated that favorable pregnancy outcomes required complement regulation at the maternal-fetal interface. In contrast to Crry, DAF deficiency in the mouse did not affect reproductive outcomes (46) suggesting DAF at the maternal-fetal interface was not critical for embryo survival. Thus, while unregulated complement activation is a threat to pregnancy, some complement components favor both the success of a pregnancy and normal fetal growth at multiple steps throughout gestation, from pre-implantation to placental formation and labor and parturition.

## Role of Complement in Pre-Implantation

**Figure 4:**
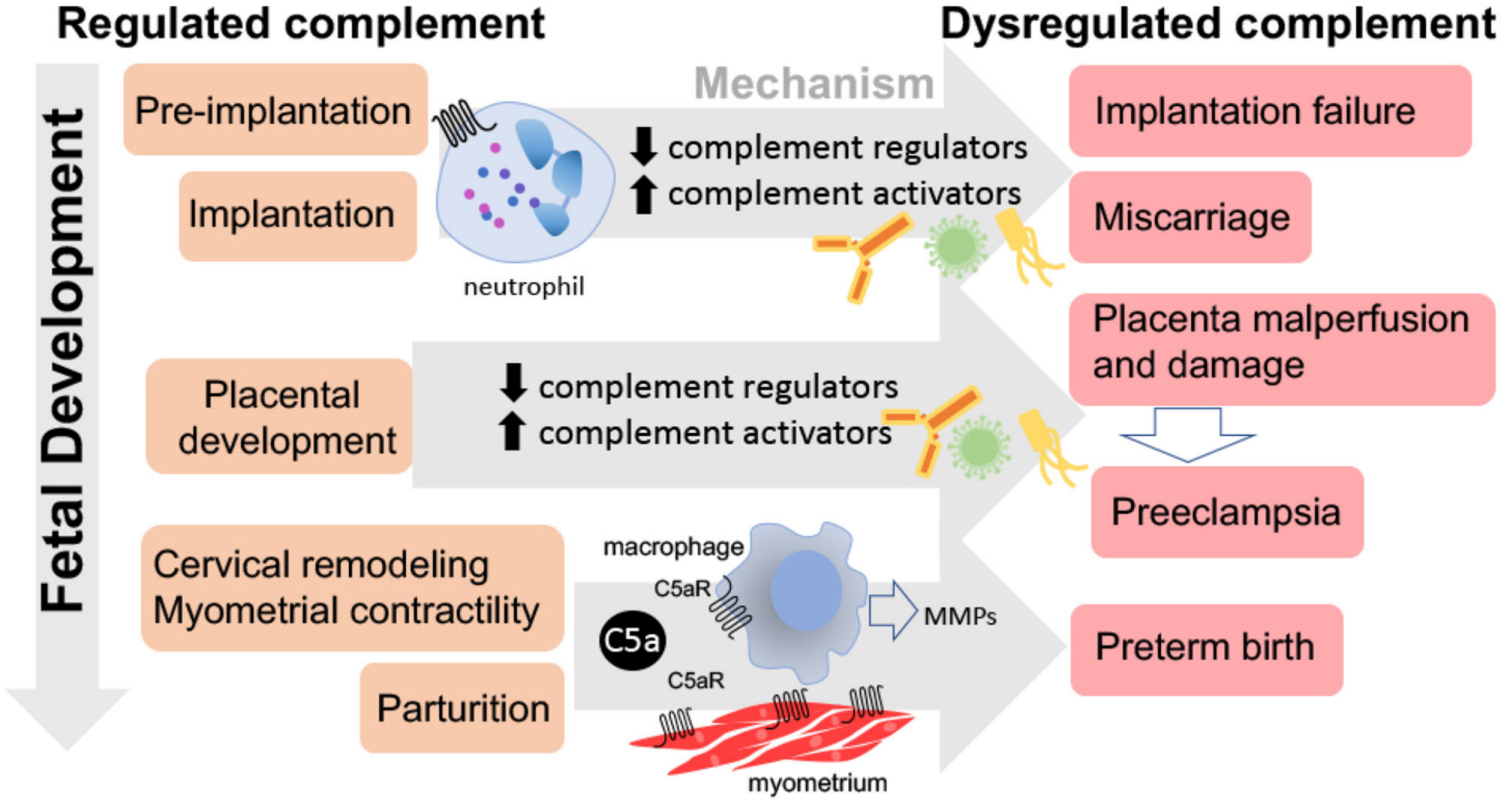
Consequences of dysregulated complement throughout pregnancy. Dysregulated complement activation from pre-implantation through parturition may lead to pregnancy complications. Excessive complement activation and neutrophil infiltration or lack of complement during pre-implantation or implantation may result in implantation failure or miscarriage. Insufficient complement regulation may lead to placental malperfusion, placental damage, and/or preeclampsia. While the C5a-C5aR axis stimulates macrophage production of metalloproteinases (MMPs) leading to normal parturition and labor, inappropriate timing may result in preterm birth.

In the early 1990s, discovery of the ability of C3b and CD46 to facilitate sperm oocyte interactions prompted numerous investigations of the importance of complement in development as reviewed in Anderson et al. (47) and Hawksworth et al. (48). In the early stages of pregnancy, the fertilized egg makes its way down the fallopian tube and into the uterus, with cell division along the way resulting in formation of 4 and 8 cell stage embryos and eventually the blastocyst. Blastocyst implantation in the uterine wall occurs at about day 9 in humans and day 4 in mice and rats (49). Complement components are found in mucosal secretions in the fallopian tubes, cervix, and uterus. Thus, as a semi-allogeneic collection of cells, the embryo is subject to complement attack before and after implantation in the uterine wall. A recent elegant study by Reichhardt et al. investigated complement targeting of the embryo prior to implantation, as well as the ability of the embryo to produce complement components (50). Cryopreserved human embryos not needed for *in vitro* fertilization were cultured to 4 or 8 cell stage and examined for expression of complement proteins and for evidence of activation of complement on their surface. Complement activation on the surface of the embryo was evident, indicating that pregnancy failure could potentially result from excessive complement activation in the pre-implantation stage (Figure 4). Inadequate or dysfunctional complement regulators could also contribute to excessive complement activation at this early stage. Reichhardt demonstrated embryonic expression of complement regulators at the pre-implantation stage that likely limit excessive activation and loss. Genetic mutations in complement regulators have been associated with recurrent pregnancy loss (51), so inadequate complement regulators at the pre-implantation stage could be a cause of pregnancy failure. In Reichhardt's study, no C5 was detected on the embryo surface, suggesting that the embryonic complement regulators were normally able to successfully limit continuation of complement activation after C3. Soluble complement regulators such as C4BP and Factor H were also evident. The CD55 and CD59 expression was seen primarily at cell junctions, suggesting a role in cellular interactions. C5 on the zona pellucida was very evident, suggesting that extensive complement activation occurred on this glycoprotein membrane surrounding the early embryo. In addition, results of this study demonstrated that oocytes and early 4 and 8 cell stage human embryos were capable of generating all of the complement components needed for C3 and C5 activation. Thus, the potential for intracellular activation of complement as well as the traditional extracellular activation is present. This allows one to speculate that intracellular signaling of complement in the embryo may play an important role similar to that demonstrated in T cells (52), where complement functions intracellularly to regulate basic metabolic processes and cellular differentiation.

The ability of the embryo to generate C3 and iC3b may play an important function in promoting embryonic growth and protecting from autophagy. C3 is an embryotrophic factor, primarily through conversion to C3b or iC3b (53–55) and likely important for embryonic growth prior to development of the placenta in both humans and rats (56). In addition, intracellular complement component C3 upregulation in human pancreatic β cells is cytoprotective and involved in autophagy regulation, contributing to β cell survival and the protection from autophagy provided by C3 in the pancreatic β cell (27). C3 may play a similar role in fetal development of the pancreas and other organs.

Endothelin is a potent vasoconstrictor that is best known for its potential role in high blood pressure. However, use of endothelin antagonists during pregnancy is contraindicated because of teratogenic effects, with known adverse developmental effects on the kidney (57). Jeoung et al. hypothesized that endothelins are required in the oviduct to facilitate early embryonic development, and they demonstrated that antagonism of endothelin resulted in a reduction in the number of two cell embryos developing (58). Our studies with the endothelin 1 antagonist, atrasentan, in the third trimester of rat gestation demonstrated that this inhibitor down-regulated the message for complement regulators in the placenta, suggesting that endothelin was important for controlling complement activation by influencing the expression of complement regulators (59). Thus, endothelin in normal embryonic development may ensure up-regulation of expression of complement system regulators to protect the embryo. One of the adverse effects of endothelin antagonists on development could be due to reducing complement regulators in the placenta or fetus, thus compromising placental and fetal development and increasing susceptibility of the placenta and fetus to attack by complement.

## Complement From Implantation Through Placental Development

A recent publication by Pierik et al. reviewed animal and human studies regarding dysregulation of complement activation in placental dysfunction and preeclampsia (60). Thus, review of that literature in this review article will not be repeated, but some notable more recent publications considered. A recent prospective study by He et al. (61) also looked at complement activation in preeclampsia across the course of pregnancy with evidence that dysregulation of the classical and alternative pathways occurred as early as the first trimester in preeclampsia, and alterations in C3a and C5a were evident throughout. Nevalainen et al. demonstrated that C7 was upregulated in severe early onset preeclampsia compared to late onset (62), demonstrating the heterogenous nature of preeclampsia and indicating that the role of complement may differ. Our continued review will focus on early events in implantation that might contribute to fetal loss, intrauterine growth restriction and placental dysfunction.

Miscarriage includes all pregnancy losses before 24 weeks of gestation. Some miscarriages occur very early at the stage of implantation and other miscarriages occur in later stages of placental development. Implantation involves trophoblast invasion of the decidua to begin the spiral artery remodeling, a critical event in normal placental development. Problems with implantation can easily cascade or present as problems with placental development and eventual placental insufficiency (63).

Mice genetically deficient in complement components have difficult or non-viable pregnancies, and as such, pregnancy failures could be due to multiple reasons. Pregnancy in the absence of C3 is one of the best studied. Mice lacking C3 have smaller blastocysts, suggesting pre-implantation events are compromised by the lack of C3, perhaps due to the lack of C3 embryotrophic properties or the importance of C3b in sperm oocyte interactions. Since C3 is an embryotrophic factor, embryonic growth prior to development of the placenta may be compromised (56). In C3 knockout mice, a normal number of implantation sites is evident at day 8, suggesting the initial implantation process is not affected (64). The placenta is fully formed by 18–20 weeks in the human and by GD11 in the rat (65). The number of implantation sites in C3 knockout mice are reduced at day 15 with more resorptions, smaller fetuses, and reduced placental size indicating the importance of C3 in normal placental development (64). Thus, the implantation sites at day 8 do not result in normal placental development by day 15, and inadequate fetal growth is realized. In humans, patients who had 3 miscarriages had higher C3 concentrations than women who successfully had a live birth after 2 miscarriages (66), suggesting that too much C3 could be detrimental just as too little C3 is associated with adverse pregnancy outcomes.

Mohlin investigated the importance of gene variants of C3 (67), as well as complement regulators CD46, CD55, and C4BP (51) in recurrent spontaneous pregnancy loss. In each case they found rare variants of the C3, CD46, and C4BP that could result in a potentially dysfunctional protein. An association with miscarriage was suggested but not significant, and needs to be evaluated in larger cohorts, similar to studies that identified complement variants important in atypical hemolytic uremic syndrome. A different study found that a polymorphism in Factor H was associated with a decrease in risk of recurrent pregnancy loss (68). Factor H in part controls complement activation on cells by binding to sialic acid. A fetus without sialic acid is not viable because of maternal complement system attack (69). Thus, lack of sialic acid would reduce the ability of Factor H to protect the fetus from complement attack, leading to uncontrolled C3 activation. If C3 is depleted in the absence of sialic acid, fetal viability is restored. In the kidney and retina, VEGF action on endothelial cells results in upregulation of Factor H and reduced complement activation. In preeclampsia, an increase in sFlt-1, a decoy VEGF receptor (VEGFR-1) is observed which would effectively reduce VEGF signaling, limit Factor H and theoretically result in increased placental complement activation. Karumanchi's group demonstrated that placental sFlt is associated with complement activation in the placenta of preeclampsia patients and could be responsible for the trophoblast damage seen (70).

C1q deficient mice have reduced litter size, suggesting a significant effect on fetal viability. This reduced fetal viability could be due to the placental insufficiency observed (71) or altered vascular function demonstrated in offspring of preeclamptic like pregnancies (72–74). In humans, another avenue of research associated with complement and recurrent pregnancy loss relates to development of anti-C1q antibodies. These antibodies are detected in lupus nephritis as well as in anti-phospholipid syndrome. C1q is critical to formation of a normal placenta (60), and the hypothesis is that interference with C1q action would interfere with placental development, but anti-C1q could also result in excessive complement activation leading to pregnancy loss. Ohmura et al. (75) conducted a very interesting study demonstrating increased anti-C1q antibodies in women experiencing recurrent pregnancy loss, but also demonstrated that anti-C1q administration to a pregnant mouse in the third trimester led to miscarriage and increased complement activation. In patients with SLE or antiphospholipid syndrome, the extent of complement activation may predict the risk of adverse pregnancy outcomes (76).

In pregnancies resulting from *in vitro* fertilization and embryo transfer, the incidence of adverse pregnancy outcomes tends to be greater. Zhao et al. (77) hypothesized that this may be due to changes in complement and/or coagulation pathways. Placentas were obtained in the first trimester following *in vitro* fertilization and embryo transfer and processed for microarray analysis. They found upregulation of numerous complement components and downregulation of regulators such as CD59, predisposing the placenta to increased complement activation compared to normal pregnancy.

A great deal of work has been done in the abortion prone mouse model CBA/J X DBA/2 mouse demonstrating complement involvement and this has been reviewed in the past (7, 78). In addition, the BPH mouse model and the pregnant Dahl SS rat represent a superimposed preeclampsia model; a mildly hypertensive animal that develops preeclamptic like symptoms when pregnant. In the BPH model, data indicate complement involvement in the implantation stage consistent with complement involvement reported in other models. However, the BPH model has not realized widespread use (45, 79–81).

## Complement in Parturition and Labor

Evidence of a role for complement in normal parturition and labor is primarily obtained from studies demonstrating a role for complement in the pathophysiology of preterm birth (PTB). Each year, almost 15 million premature children are born worldwide. Complications of PTB are the leading cause of death in children younger than 5 years of age worldwide (82), and premature infants are particularly vulnerable to brain injury. Increasing evidence suggests that labor and delivery are triggered by inflammatory reactions including the complement system (83). Mouse and human studies underscore the role of complement activation in the initiation of labor, cervical remodeling as well as in uterine contractions. Two mouse models of PTB have provided evidence for the importance of complement activation in cervical remodeling and PTB (Figure 4). In one model, PTB was induced by vaginal administration of lipopolysaccharide (LPS) to mimic one of the most common clinical scenarios of ascending infection and inflammation (84). In the other model, PTB was induced by administration of progesterone antagonist RU486 that induces inflammation leading to cervical ripening in mice and women (84, 85). Results in both models demonstrated increased cervical C3 deposition, macrophage infiltration, and serum C3a des Arg and C5a des Arg levels in PTB when compared to gestational age-matched controls.

Results in both models of PTB demonstrated increased cervical distensibility with histological studies revealing a significant degradation of collagen and increased matrix metalloproteinase 9 (MMP-9) activity in the cervix (Figure 4). However, neither LPS nor RU486 treatment caused increased MMP-9, cervical remodeling or PTB in C5aR deficient mice. These data indicate that C5aR is required for the cervical remodeling that precedes PTB (84). In response to C5a or cytokines, macrophages release MMP-9 leading to collagen digestion, cervical ripening, and increased distention leading to preterm parturition. Progesterone is one of the few treatments available to prevent PTB in women with short cervix. Vaginal progesterone both decreases the risk of preterm birth and improves perinatal outcomes, with no apparent adverse effects on childhood neurodevelopment (86). Interestingly, animal studies suggest that the protective effects of progesterone might be related to the complement system. Progesterone reduced C5aR on the macrophage surface, inhibited the release of MMP-9, reduced cervical remodeling and prevented PTB (84). In addition, in LPS treated mice, depleting macrophages also prevented cervical remodeling and PTB. Also, C5a-C5aR interaction was required for MMP-9 release from macrophages (84) as well as for the cervical remodeling that leads to PTB, suggesting that complement inhibition may be a therapeutic option to prevent this serious pregnancy complication. Patients with paroxysmal nocturnal hemoglobinuria (PNH) have defective complement regulators, CD55 and CD59, and an increased incidence of adverse pregnancy outcomes. The anti-C5 antibody, eculizumab is used to control the RBC hemolysis in these patients with good outcomes (87). Eculizumab has also been used in PNH patients during pregnancy with favorable pregnancy outcomes (88). However, controlled studies are needed to determine if eculizumab affects incidence of PTB in pregnancies of mothers with fully functional complement systems.

The transformation of the cervix from a closed rigid structure to one that relaxes sufficiently for birth i.e., cervical ripening, depends at least in part on C5a-C5aR. This dynamic process begins long before the onset of labor and must be synchronized with uterine contractions to propel the fetus out of the uterus (expulsion stage). Interestingly, a role for complement activation in myometrial contractions was also demonstrated (89) *in vitro* in mouse and human myometrium. Increased C5a was detected in the myometrium of mice that received intravaginal LPS to induce preterm birth but not in myometrium from age-matched controls or myometrium harvested at term. In human and mouse isolated uterine myometrium, C5a increased contraction frequencies and expression of connexin 43 (Cx43) suggesting that C5a is a uterotonic molecule. Cx43 is a myometrial contraction-associated protein involved in uterine contractility and onset of labor (89). Pravastatin prevented cervical remodeling, myometrial contractions, and preterm labor in a mouse model of PTB (89), and also increased the synthesis and expression of DAF in the cervix, thus inhibiting complement activation (89). These data suggest that statins may be beneficial in complement-mediated pregnancy complications.

Molecules of the innate immune system termed collectins include surfactant proteins SP-A, SP-D, and mannan-binding lectin (MBL). Collectins are found in amniotic fluid and at the maternal-fetal interface. SP-A, SP-D, and MBL reach maximum concentrations at term in amniotic fluid, suggesting they may play a role in pregnancy maintenance and parturition. Other studies suggest that SP-A and SP-D are involved in onset of labor. The recombinant forms of SP-A and SP-D increased CX43 expression and contraction of a human myometrial cell line, ULTR, when grown on collagen matrices (90). In addition, SP-A and SP-D increase the expression of proinflammatory cytokines, IL-8 and IL-6, that are found in high concentrations in serum from women with increased risk of spontaneous preterm birth (91).

SP-A expression in mouse fetal lungs and its secretion in amniotic fluid represents a signal for the onset of parturition (92, 93). In pregnant mice, injection of purified SP-A into the amniotic fluid stimulates IL-1β production and subsequent preterm delivery. These results were further verified by injecting antibodies to SP-A into the mouse amniotic sac leading to a delay in parturition (92). These studies provide evidence that SP-A and SP-D play an important role in modulating events prior to labor by inducing the synthesis of myometrial contraction-associated proteins and pro-inflammatory cytokines changing the quiescent uterus to a contractile uterus.

Multiple studies in humans underscore the important role of complement activation in the pathogenesis of PTB. Women with increased complement factor Bb in early pregnancy were 4-fold more likely to have PTB compared to women with lower levels (94). In the absence of intraamniotic infection, preterm parturition increases plasma concentrations of complement fragment Bb (95). Interestingly, this activation does not occur in spontaneous labor at term suggesting that the mechanisms leading to pre-term and term labor fundamentally differ with regards to a role for complement activation. This is consistent with mouse studies of PTB (96). In addition, elevated levels of C3a in the first trimester of pregnancy are predictive for PTB and premature rupture of membranes (PPROM) (97). A follow-up study by the same group found higher concentrations of C3a in PTB cases compared to term controls, reinforcing the concept that complement plays a role in the pathogenesis of premature delivery (94). Finally, elevated concentrations of complement factors C3a, C4a, C5a, and Bb have been detected in the amniotic fluid of women with PTB with microbial invasion of the amniotic cavity (98).

Recent studies of the microbiome in pregnancy found that the vaginal bacterial taxonomic composition might be associated with the time of delivery. Lactobacillus-deficient vaginal communities and elevated *Gardnerella* and *Ureaplasma* species were associated with a higher risk of PTB (99). Dysregulation of complement system by a specific uterine microbiome may lead to infection and PTB. It is also proposed that additional pathogenic bacterial species which are not detectable by traditional culture-based methods may initiate complement dysregulation and produce inflammatory mediators to cause cervical remodeling, increased uterine contractility, and increasing the risk for preterm birth. Supporting the role for pathogens in the onset of PTB, intrauterine infection is a definitive risk factor for PTB. However, targeting infection with the use of antibiotics has not reduced the risk of PTB. One potential cause of the antibiotic failure is the polymicrobial flora in the reproductive tract with unknown virulence, susceptibility and antimicrobial resistance (100).

Overall, most of the evidence for the involvement of complement in preterm birth comes from studies in mouse models where preterm birth is induced by a bacterial product, LPS, or by the progesterone antagonist. Clinical studies of women experiencing PTB reinforces the mouse data. Studies have focused on the collectins SP-A and SP-D as well as the complement activation products, without any clear studies in humans delineating whether the complement activation products are an indicator of the problem, or a cause of the PTB. Studies connecting PTB with the microbiome are promising and suggest that manipulations of the microbiome may be fruitful, but much more needs to be learned about the competitive interactions of bacterial species and the influence on pregnancy.

### Role of Complement in Fetal Brain Injury in Preterm Birth

Premature babies are particularly vulnerable to brain injury. As previously described, premature labor has many hallmarks of an exaggerated inflammatory response including complement activation. Therefore, we speculate that the inflammatory mediators that induce cervical ripening and myometrial contractions, including complement cleavage products also affect fetal brain development. Animal models characterized fetal brain injury that was associated with inflammation-induced preterm birth and revealed deleterious effects on fetal brain morphology and function. Indeed, a mouse model of inflammation-induced PTB not only demonstrated signs of cortical brain injury but also demonstrated a crucial role for complement activation in this injury (101). Specifically, complement component C5a, involved in cervical remodeling and myometrial contractions was also involved in fetal brain injury, particularly, cortical brain damage (84, 89, 101). Abnormal cortical development can result in the long-term cognitive, behavioral, attentional or socialization deficits observed in children born preterm. Disruption of cortical neuron cytoarchitecture, characterized by shorter dendrites and axons, was observed in PTB-mice (101), and C5aR (C5aR^−/−^) deficiency protected fetuses from this cortical brain damage (101). Treatment with antibody to C5 preventing generation of C5a also prevented cortical fetal brain injury, providing further evidence for a role for complement.

The detrimental effects of C5a on fetal cortical neuron development and survival has also been demonstrated *in vitro*. Glutamate is the primary excitatory neurotransmitter in the brain, and excess glutamate can cause excitotoxicity and brain injury. C5a caused increased glutamate release in fetal cortical neurons in culture (101), and blockade of C5aR not only prevented the glutamate increase but also restored dendritic and axonal growth and survival. *In vivo* studies using non-invasive proton magnetic resonance spectroscopy imaging demonstrated increased glutamate in PTB-fetuses compared to age-matched controls (101). If glutamate receptors were blocked, adverse effects of C5a on isolated fetal cortical neurons were prevented, confirming that the neurotoxic effects of C5a on the fetal brain are mediated by glutamate (101). Interestingly, increased C5a is found in the cerebrospinal fluid of newborn human infants born preterm compared to those that were born at term and these observations were independent of systemic infection (102).

## Complement in Fetal Development

### Brain Development

**Figure 5:**
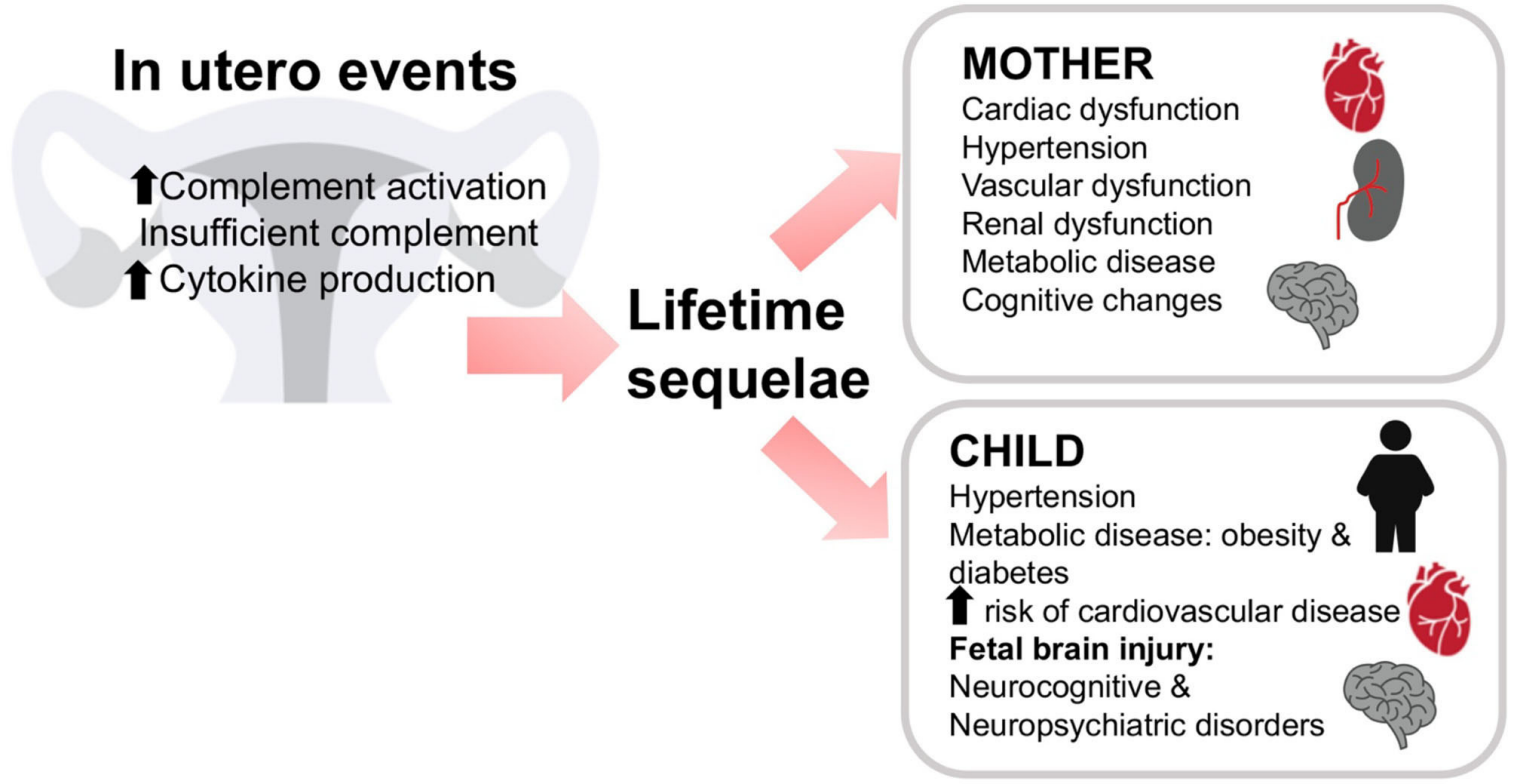
*In utero* events can have lifelong consequences. Insufficient or dysregulated complement may result in lifelong sequelae in both mother and child. Either a lack of complement activation or excessive activation of complement or cytokine production *in utero* may lead to problems throughout the life of the mother or child. The mother may experience cardiac, vascular, or renal dysfunction, hypertension, metabolic disease, or cognitive changes. The child may also experience hypertension, metabolic disease, diabetes, or cardiovascular disease. In addition, fetal brain injury may result in cognitive and/or psychiatric disorders in the offspring.

In addition to its well-documented role in immune surveillance and host defense, the complement system plays other roles in the central nervous system. Like cells in many other organs, brain cells can produce complement proteins and receptors (103, 104). Similar to the placenta, the role of the complement system in the brain is described as a double-edged sword (105). Complement activation can result in either protective or deleterious effects on the brain (106). On one hand, complement proteins and receptors aid in brain developmental processes such as neurogenesis, neuronal migration and synaptic remodeling (107–109). On the other hand, deleterious effects of complement activation have been observed in the developing fetus resulting in neurocognitive and psychiatric disorders [Figure 5; (110–112)]. Maternal hypertension or preeclampsia that is associated with increased complement activation also significantly increases the risk of mental disorders in the offspring (113).

C5a–C5aR1 signaling plays a functional role in mammalian neurogenesis (114). During mouse embryogenesis, C5a signaling increases proliferation of neural progenitor cells in the ventricular zone that are required for normal brain development. Importantly, inhibition of C5aR1 in human and mouse models reduced proliferation and symmetric division of apical neural progenitors, demonstrating the crucial role of C5a- C5aR1 signaling in brain development (114). Proper brain formation and establishment of neural circuits requires neuronal migration. During development, excitatory neurons arising from the ventricular zone undergo radial migration to reach their correct laminar position in the cortex (109). This process appears regulated at least in part by the Lectin pathway. Mice deficient in C3, Masp1, or Masp2 exhibit impaired radial migration resulting in improper positioning of neurons and disorganized cortical layers (107). Interestingly, polypeptides that mimic C3a or a dual C3aR/C5aR agonist partially rescued the deficits in migration in C3 or Masp2-deficient mice. This suggests that activation of the Lectin pathway leading to C3a/C5a generation and activation of both C3aR and C5aR are critical for radial neuronal migration and cortical development (109). However, the role of the complement system in particular C5a in neuroinflammation, and neurodegeneration has also been demonstrated (114). The abnormal fetal brain development observed in a mouse model of PTB characterized by increased levels of C5a suggests that exposure to increased complement activation *in utero* can also disrupt fetal brain development (101).

The timing and the degree of the stimuli leading to complement activation during fetal brain development is crucial in distinguishing between protective and harmful effects. The blood–brain barrier (BBB) is instrumental in limiting passage of cells and large molecules from the blood into the brain (115). Brain endothelial cells are exposed to complement proteins from the circulation as well as local brain synthesis, and these complement proteins and activation products can result in increased permeability of the BBB (116). The BBB permeability increases after C5a signaling (117). Similarly, maternal overexpression of cytokines and complement activation products observed during complicated pregnancies may increase the permeability of the fetal BBB allowing the invasion of peripheral blood-derived inflammatory cells and molecules, including complement proteins, leading to fetal brain injury (118). In addition, complement split product C5a might induce microglial inflammatory polarization in the fetal and neonatal brain. Activation of microglia has been associated with neurogenic hypertension and behavioral abnormalities in the offspring (72, 119).

The autoimmune disorder antiphospholipid syndrome (APS) is characterized by vascular thrombosis and/or adverse pregnancy outcomes in combination with circulating antiphospholipid antibodies. Over the past two decades, a critical role for complement activation emerged in the pathogenesis of pregnancy complications in APS in women and animal models (76, 120–124). For example, increased fetal brain C3 deposition and robust maternal complement activation occurred in a mouse model of obstetric antiphospholipid syndrome (OAPS) induced by the passive transfer of human antiphospholipid antibodies into a pregnant mouse (123). Ultrasmall paramagnetic iron oxide (USPIO) particles conjugated to monoclonal antibodies against complement C3 split products (C3b, iC3b, and C3c) are a useful tool to detect complement activation *in vivo in utero* by magnetic resonance imaging (MRI) in mice (110). Using this non-invasive technique, increased fetal brain C3 deposition was observed in OAPS-mice and was associated with disruption of the cortical axonal cytoarchitecture as well as increased neurodegeneration (110). Interestingly, C3 deposition in fetal brains in OAPS was also associated with diminished levels of glucose, lactate, and choline derivatives; molecules involved in energy metabolism, membrane lipid function, and neuroprotection (125). Increased placental C3 was also detected in OAPS-mice, using MRI (110). These observations are in agreement with human studies showing complement deposition in the placentas of women with OAPS (121).

Treatment with complement inhibitor hydroxychloroquine (HCQ) protected fetal brain development and prevented fetal brain metabolic abnormalities in OAPS-mice (125). C3 deposition detected in the fetal brains in OAPS-mice was associated with anxiety-related behavior after birth. The decrease in open field and open arm activity in the elevated plus maze (duration and/or entries) observed in the offspring of the OAPS-mouse indicates an anxiety-related behavior. In this line, the complement system is linked to developmental brain disorders, resulting in neuropsychiatric disorders such as schizophrenia (126) and autism spectrum disorder (127).

Similar to the OAPS mice, the offspring from mice infected with malaria showed abnormal neurodevelopment and neurocognitive impairment characterized by abnormal learning and memory and depressive-like behavior compared to controls (110). Interestingly, if the C5aR was deleted in the fetuses or infected pregnant mice were treated with anti-C5 antibody, the neurocognitive impairments of malaria-exposed offspring were prevented. This is similar to results in the PTB-model (101, 111). These studies clearly demonstrate a role for complement component C5a in cortical brain injury and associated behavioral abnormalities observed in fetuses exposed to excessive intrauterine inflammation.

Maternal infections and other pregnancy complications are a major risk for the development of neonatal hypoxic ischemic (HI) encephalopathy, a major cause of neonatal mortality and morbidity (128) with limited clinical options for treatment. Animal studies indicate that deficiency of Properdin in the neonatal HI brain is neuroprotective (128), suggesting that Properdin could be a therapeutic target to limit neonatal brain injury.

Clearly normal fetal brain development requires a functioning complement system for neuronal cell migration and synaptic pruning. Thus, any manipulations of complement during pregnancy must consider the stage of fetal development *in utero* and track consequences to brain health in the offspring. Much has been learned about a common role for the complement system in neurodevelopmental disorders in pregnancy using two different mouse models of adverse pregnancy outcomes: obstetric antiphospholipid syndrome as well as placental malaria. This provides evidence that limiting excessive complement activation *in utero* due to multiple causes may be valuable in minimizing neurodevelopment and neurocognitive impairment in complicated pregnancies.

### Heart Development

Congenital heart block (CHB) accounts for nearly 30% of all major congenital anomalies (129). Recent studies showed that mutations in the immune-related molecules mannan-binding lectin (MBL)-associated serine protease (MASP)-3 underlie the etiology of congenital heart block (130). MASP activation resulted in complement deposition and inflammatory cell infiltrates in hearts of neonates who died from CHB (131), suggesting that complement activation is a mediator in the fetal cardiac tissue damage. Autopsy specimens from babies with CHB showed deposition of immunoglobulin and complement components in all cardiac tissues, reinforcing the pathogenic effects of complement in CHB (132).

## Complement Activation During Pregnancy and Preeclampsia: Long Term Consequences For Mother and Child

Both animal and human studies demonstrated the importance of complement activation in the pathogenesis of preeclampsia. In humans, mutations in complement protein and complement regulatory protein genes lead to increased susceptibility to preeclampsia (133, 134) and others suggest that complement cleavage products C3a and Bb can serve as predictors of preeclampsia (94, 135). Preeclampsia has historically been considered a transient condition since acute maternal symptoms resolve with delivery of the placenta. However, preeclampsia is not an isolated disease of pregnancy but results in long-term renal and cardiovascular disease associated with a history of maternal preeclampsia (Figure 5). In 2011, the American Heart Association included preeclampsia as a gender-specific risk for cardiovascular disease (136). Offspring of pregnancies affected by preeclampsia also have an increased cardiovascular risk profile. Increased blood pressure and body mass index are evident in children and young adults born to pregnancies complicated by preeclampsia (137). Therefore, it is now well-accepted that preeclampsia is linked to an array of maternal morbidities that occur later in life as well as long term adverse health effects in the offspring. However, the mechanism behind the maternal and offspring health complications after a preeclamptic pregnancy remain unknown. It is possible that abnormal placentation during preeclampsia results in placental insufficiency leading to the release of vasoactive and proinflammatory molecules that compromise the maternal and fetal health after pregnancy (72).

Maternal and umbilical cord plasma C5a concentrations are significantly higher in a preeclamptic pregnancy than in normotensive pregnancy (138). That maternal and cord plasma C5a concentrations directly correlate suggests that C5a freely moves between the maternal and fetal circulation (138). In addition, C5a interaction with C5aR on trophoblasts releases anti-angiogenic factors that impair normal placentation leading to preeclampsia and the associated placental insufficiency (139). Finally, C5a levels in women with preeclampsia positively correlate with the maternal autoantibody to the angiotensin Type 1 receptor, a potential contributor to the pathogenesis of preeclampsia (140).

In the mouse, a paternal deficiency of C1q results in impaired trophoblast migration and abnormal placentation leading to onset of preeclampsia-like symptoms including endothelial dysfunction and hypertension in the mother, along with high levels of C5a (72, 141, 142). Offspring of these pregnancies also experienced health complications (Figure 5). Glomerular injury persisted after the preeclampsia-like pregnancy, leading to fibrosis. In addition, left ventricular remodeling with increased collagen deposition and MMP-9 expression and enlarged cardiomyocytes developed after the preeclampsia-like pregnancy (72). Hearts were characterized by increased left ventricular internal wall thickness and mass, increased end diastolic and end systolic volumes, and increased stroke volume. Placenta-derived bioactive and proinflammatory factors (endothelin-1, IL-6, and C5a) increased in maternal sera during and after a preeclamptic pregnancy. Offspring of preeclamptic mice developed endothelial dysfunction, hypertension, and indicators of metabolic disease (72). Pravastatin treatment normalized C5a values in preeclamptic-mice and normalized cardiovascular and metabolic function in both mothers and offspring. This suggests that C5a elevation in this preeclampsia model of placental insufficiency resulted in long-term, cardiovascular and metabolic effects in the mother and offspring. Interestingly, increased C5a also occurs clinically in mother and offspring during and after preeclampsia (137).

Mechanically-induced placental ischemia in the third trimester of pregnant rats results in increased complement activation and hypertension. Inhibiting complement activation attenuates the hypertension, suggesting that complement is important (143). Offspring of these pregnancies also exhibit fetal growth restriction, high blood pressure, and glucose intolerance (144, 145) as well as reduced pancreatic β cell area (146). A role for the complement system in these events in the offspring has not been investigated to date. However, given that decreased C3 is associated with reduced pancreatic beta cell area in the adult with the potential for increased risk of Type 2 diabetes, further investigation of complement involvement in pancreatic development is certainly warranted (27).

Growing evidence indicates the importance of passage of maternal-derived mediators across the placenta to the fetus. These mediators include complement activation products that freely cross the placenta and reach the fetus affecting its cardiovascular and nervous system (Figure 5). This is in agreement with the “developmental origins of adult disease” hypothesis, which proposes that disease risk as an adult is determined by prenatal exposures (147). We previously described how exposure *in utero* to placental pathogenic mediators such as C5a might affect fetal neurodevelopment leading to abnormal fetal brain cytoarchitecture and abnormal behavior in the offspring (110). Microglia, resident brain macrophage cells express C5a receptors and therefore maternal-derived C5a may activate the cells. Microglia activation increased in the neonatal brains in the mouse preeclampsia model (72). Activated microglia can lead to neurogenic hypertension in the offspring and cognitive disorders (119).

Preeclampsia affects multiple organ systems, including the maternal brain. While cerebrovascular dysfunction during preeclampsia, leading to cerebral edema, seizures and stroke has been extensively characterized, the potential long-term effects of preeclampsia on neurocognitive functions and behavior are not well-understood (148). Some women with a history of preeclampsia reported cognitive and emotional changes during the postpartum period and subsequent years (149). While objective data supporting these findings are very limited, it is tempting to speculate that in a similar manner to the fetal brain, placental-derived factors might reach the maternal brain, and exert harmful effects. This might be aided by the increased permeability of the maternal BBB caused by proinflammatory molecules released during preeclampsia and other pregnancy complications associated with an excessive proinflammatory response.

## Perspectives for Future Research

The continual challenge with evaluating the role of the immune system, including the complement system, in pregnancy is a determination of too much or too little. Insufficient complement system involvement as well as too much complement system involvement can lead to pathophysiology. Thus, determining the proper balance in each organ or disease state is required. This balance point differs over the course of a pregnancy, as well as the developmental stage of the organism. In addition, the complexity increases in terms of deciding whether increased complement involvement is the cause or a consequence of the problem i.e., intended to clean up or resolve the pathophysiology. Manipulation of complement in the normal state may have adverse effects, whereas manipulation of complement in the diseased state may be beneficial.

Our review of complement in pregnancy has suggested some gaps in the literature in terms of pre-implantation, preterm birth, induction of labor, and adverse effects in offspring following a preeclamptic pregnancy. The investigation of the role of complement regulators at points of cell interactions in pre-implantation embryos could further illuminate the homeostatic function of complement in development in organ systems other than the brain. The positive effects of eculizumab in treatment of pregnant women lacking complement regulators suggest that controlled studies are needed to determine if eculizumab affects incidence of PTB in pregnancies of mothers with fully functional complement systems. In addition, pre-clinical studies suggest that evaluating properdin or other therapeutic modulators of complement may decrease PTB and the ensuing developmental side effects in offspring. Additional studies with larger sample sizes are needed to examine variants of complement inhibitors as well as to determine the risk factors of adverse pregnancy outcomes. Further studies investigating interactions of the complement system and specific microbiome components which negatively influence a successful pregnancy are also needed. Studies assessing the role of surfactant proteins SP-A and SP-D in induction of labor make it clear that the role for another collectin, mannan-binding lectin (MBL) needs to be investigated since it also reaches a maximum concentration at term. In the absence of intra-amniotic infection, preterm parturition increases plasma concentrations of complement fragment Bb. Interestingly, this activation does not occur in spontaneous labor at term suggesting that the mechanisms leading to pre-term and term labor fundamentally differ with regards to a role for complement activation. This is consistent with mouse studies of PTB which could be used to clarify the distinct mechanisms. Of course, complement effects *in utero* can have major developmental effects after birth in the offspring as clearly indicated by studies of brain development. These types of studies need to be extended to pathophysiology and incidence of other disorders associated with preterm birth, preeclampsia, intrauterine growth restriction such as hypertension, metabolic disorders, obesity, and Type 2 diabetes. Given that decreased C3 is associated with reduced pancreatic beta cell area in the adult with the potential for increased risk of Type 2 diabetes, further investigation of complement involvement in pancreatic development is certainly warranted (27), as is the connection between complement and obesity which increases risk for multiple adverse pregnancy outcomes.

## Conclusion

While the role of complement in the pathogenesis of pregnancy complications such as miscarriage, preeclampsia and preterm birth has been supported by substantial evidence, new studies demonstrate that the complement system is also involved in the early steps of pregnancy such as conception and embryo implantation. Even more, the effects of complement activation seem to go beyond pregnancy and have long term effects. Exposure to excessive complement activation during pregnancy clearly has deleterious effects after pregnancy, both on the health of the mother as well as the offspring. Although additional studies are required, modulation of complement may have important ramifications, both beneficial and harmful, from pre-implantation through the lifetime of the offspring.

## Author Contributions

GG, JR, JL, and SF wrote the review article. GG prepared the figures. All authors reviewed the manuscript and approved the submitted version.

## Conflict of Interest

The authors declare that the research was conducted in the absence of any commercial or financial relationships that could be construed as a potential conflict of interest.
